# Epidemiology of intussusception before and after rotavirus vaccine introduction in Fiji

**DOI:** 10.1038/s41598-018-29515-2

**Published:** 2018-07-25

**Authors:** Felisita Tupou Ratu, Rita Reyburn, Evelyn Tuivaga, Asena Tuiketei, Kylie Jenkins, Kim Mulholland, Adam Jenney, Fiona Russell

**Affiliations:** 1 Ministry of Health and Medical Services, Suva, Fiji; 2 Murdoch Children’s Research Institute, Melbourne, Victoria, Australia; 3 Fiji Health Sector Support Program, Suva, Fiji; 4 Telethon Kids Institute, Perth, Western Australia Australia; 5 Menzies School of Health Research, Darwin, Northern Territory Australia; 6 London School of Hygiene and Tropical Medicine, London, UK; 7 Fiji National University, Suva, Fiji; 8 Centre for International Child Health, Dept. of Paediatrics, The University of Melbourne, Melbourne, Victoria, Australia

## Abstract

In 2012, Fiji introduced rotavirus vaccine (Rotarix, GSK) into the national immunisation schedule. We describe the intussusception epidemiology prior to rotavirus vaccine, temporal association of intussusception cases to administration of rotavirus vaccine, and estimate the additional number of intussusception cases that may be associated with rotavirus vaccine. A retrospective review of intussusception cases for children aged <24 months old was undertaken between January 2007 and October 2012 pre-vaccine. All admissions and deaths with a discharge diagnosis of intussusception, bowel obstruction, paralytic ileus, or intussusception ICD10-AM codes were extracted from national databases and hospital records. Nationwide active intussusception surveillance was established for three years post-vaccine (2013–2015). There were 24 definite intussusception cases in the pre-rotavirus vaccine period, 96% were confirmed by surgery. The median age was 6.5 months. The incidence rate was 22.2 (95% CI: 13.9–33.7) per 100,000 infants. There were no deaths. Active surveillance identified 25 definite intussusception cases, 96% of which were among children who were age-eligible for rotavirus vaccine. None were potentially vaccine related. We estimated one to five additional  cases of intussusception every five years. The incidence of intussusception pre-rotavirus vaccine in Fiji is low. Intussusception associated with rotavirus vaccine is likely a rare event in Fiji.

## Introduction

Naturally occurring intussusception (IS) occurs in infants typically aged four to 10 months, when one part of the intestine telescopes into the adjacent section of the intestine. Documented baseline of IS rates vary by population and is attributed to number of factors such as access to healthcare, ethnicity, cultural or environmental factors^1–3^. Studies from middle- and high-income countries show the incidence rates to be 30–70 cases per 100,000 infants. In contrast, studies in Japan and Vietnam have shown substantially greater rates of IS at 185 and 302 cases per 100,000 infants, respectively^1,4^. The case fatality ratio (CFR) of IS is <1% in developed countries and varies between 1–9% in two studies conducted in Asia and Africa^1^. These differences are likely due to accessibility to health care and the availability of quality medical and surgical care^1^.

Rotavirus (RV) vaccine protects against RV diarrhoea, the most common cause of diarrhoea and severe dehydration in children worldwide^5^. The first RV vaccine Rotashield, was removed from the USA market in 1999 due to its association with intussusception (IS)^6^. Since then there has been two other RV vaccines, Rotateq (Merck) and Rotarix (GSK), which have been developed^7^. Several observational studies have shown that these RV vaccines are associated with a small increased risk of IS, with the estimate ranging between 1–6 additional IS cases per 100,000 infants vaccinated^8–10^. Due to the benefit of RV vaccination that far outweighs this risk^11^, there is continued recommendation to continue with vaccination. Currently, 80 countries include RV vaccine in their national immunisation schedules^12^. WHO now advises to monitor IS following the introduction of RV vaccine in a new population^13^.

In October 2012, Fiji introduced the monovalent RV vaccine, Rotarix (GSK) into the national immunisation schedule at six and 14 weeks of age. Vaccination data was recorded on the child health card and the health centre registers at the time of vaccination as part of the routine Ministry of Health and Medical Services (MoHMS) data collection. Due to the very small risk of developing IS following RV vaccine, we describe the IS epidemiology prior to RV introduction, the temporal association of IS cases to the administration of RV vaccine following the national introduction of RV vaccine, and estimate the additional number of IS cases that may be associated with RV vaccine in Fiji.

## Results

### IS epidemiology pre-RV vaccine introduction

**Figure 1:**
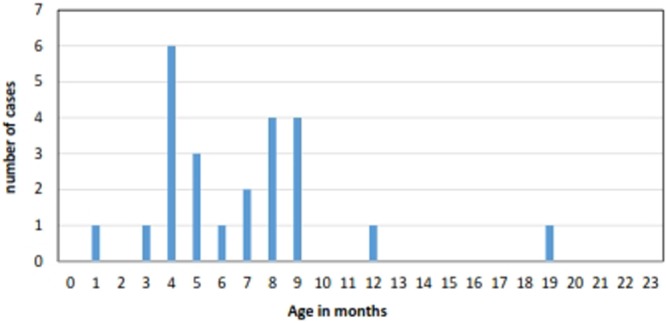
The age distribution of IS cases in children <24 months old, Fiji, pre- RV vaccine introduction, January 2007- October 2012 (n = 24).

In the pre-RV vaccine period (January 2007-October 2012) there were 35,952 hospital admissions among children under two years of age of which 64 were identified as cases of suspected IS. Of these, 24 were classified as definite IS of which 23 (96%) were confirmed at surgery (Table 1). Of the definite IS cases 21 (95%) were between three and 12 months of age (Fig. 1 and Table 1) and 11 (46%) were male (Table 1). All but one of the cases were surgically corrected, one resolved following an enema and there were no deaths (Table 1). The incidence rate of definite IS among infants and all children under two years of age was 22.2 (95% CI: 13.9–33.7) and 12.1 (95% CI: 7.7–18.0) per 100,000 population respectively.

**Table 1:** Characteristics of IS cases under two years old, pre-RV vaccine introduction, January 2007- October 2012, Fiji (n = 24).

Characteristics	Summary statistic
Median age in months (IQR)	6.5 (4–8.5)
Ethnicity, n (%)	
iTaukei	16 (70)
Fijian of Indian descent	6 (26)
Other	1 (4)
Unknown	1 (4)
Male, n (%)	11 (46)
Treatment, n (%)	
Surgery	23 (96)
Liquid contrast enema	1 (4)
Ultrasound with reduction	0
Median length of stay in days (IQR)	6 (4–8.5)
Case fatality, n (%)	0

### Population at risk post-RV vaccine introduction

Using MOHMS estimates of coverage of at least one dose of vaccine among children of eligible age there were an estimated 47,790 infants at risk of RV vaccine related IS between January 2013 and December 2015. Applying the risk of 1–6 IS additional cases per 100,000 infants to the Fijian population, would result in an estimated 1–5 cases of IS attributable to RV vaccine every five years.

### Temporal association of definite IS to the RV vaccine

During the active surveillance period, January 2013 to December 2015, there were 12,242 hospital admissions among children under two years of age of which 47 were identified as cases of suspected IS. Of these, 25 were classified as definite IS. Twenty four (96%) definite IS cases were among children who were age-eligible for the RV vaccine. Of the 24 definite IS cases whom were vaccine eligible none were categorised as potentially or likely vaccine related: Thirteen cases (54%) received RV vaccine (2 doses) >21 days pre-event (range; 86–572 and 28–516 days post the first and second dose respectively); one (4%) received the second dose of rotavirus vaccine 13 days pre-event; eight (33%) cases had unknown vaccination status but all occurred >21 days post-vaccine had they received their vaccine according to schedule at 10 and 14 weeks (age range; 18–32 weeks old); and two (9%) were unvaccinated.

## Discussion

This is the first study from a Pacific island country to describe IS following RV vaccine introduction. It is unlikely any case of IS occurred three years post-RV vaccine in Fiji. However, any additional case of IS that may occur post RV vaccine is likely to be a very rare event in Fiji and the benefits of RV vaccine outweigh any risks. These findings provide evidence of RV vaccine safety in Fiji and support the continued use of the RV vaccine in Fiji as well as its introduction into similar counties in the Asia-Pacific region.

The incidence of IS in Fiji are at the low compared to other countries in the region pre-RV vaccine introduction, with IS rates ranging from 9 to 328 cases per 100,000 infants in Bangladesh and South Korea, respectively (Table 2). The median age at which IS occurred in Fiji was 6.5 months (IQR: 4.0–8.5) which is typical for naturally occurring IS. A study in New Zealand prior to RV vaccine introduction found an adjusted incidence rate of 56 per 100,000 child-years (95% confidence interval (CI) 42–71). The study also found that there was no difference in IS incidence between ethnic groups, although cases occurred at a younger age in Māori and Pacific islander infants compared to Asian and other ethnicities (Pacific median 7.5 months (IQR 5.9–11.6), Māori 7.8 months (IQR 5.5–12.3), European 9.2 months (IQR 5.8–15.8), other ethnicity 10.2 months (IQR 8.2–12.3), Asian 10.5 months (IQR 7.0–17)^14^. However, in Taiwan, Japan, Vietnam and Korea the rates of IS hospitalisation among infants prior to introduction of RV vaccine was significantly higher with an annual incidence rate of 75, 185, 296 and 328 per 100,000 infants, respectively^1,15,16^. The reason for these higher rates in Asian infants is unknown.

**Table 2:** Incidence rates of intussusception in children <1 year in the Asia-Pacific region, by country.

Country	IS incidence rate per 100,000 children <1 yr (95%CI where available)
Bangladesh^1^	9
New Zealand^14^	56 (42–71)
Taiwan^16^	75
Australia^11^	81
Japan^1^	185
Vietnam^1^	302
Republic of Korea (South Korea)^1^	328

Although we did not have the RV vaccination status of all IS cases post RV vaccine introduction, it is unlikely that any of the IS cases were temporally associated to the RV vaccine. Most cases occurred outside of the period of risk for developing vaccine related IS had they received their first and second RV vaccine according to schedule at 6 and 14 weeks, respectively. One case occurred 13 days after the second RV vaccine dose and did not fit any category of RV vaccine associate IS case definition. The US studies to assess the temporal risk of IS into more hospitalised cases at population level, before and after the RV vaccine introduction showed an increased risk of IS hospitalisation rates for children aged eight to 10 weeks post-dose one which is consistent with recent US post-licensure studies^17^. A study in Japan, which has a high reported IS rate, found that there was a marked increase in IS cases within seven days following the first-dose of RV vaccine one, but not following the second dose of RV vaccine which is consistent with other large post-marketing safety studies globally^10,18,19^. A study in Singapore showed the risk of IS increases by roughly 8-fold during the one to seven day period following receipt of the first dose of RV vaccine in infants of Chinese, Malay, and Indian ethnicity. The relative risk of IS post-RV vaccination was not higher in Singapore despite differences in background IS incidence compared with US and Australia, or older age of vaccination^20^. Studies among Mexican and Brazilian infants showed an increased risk of IS during the one to seven days period following the first dose and second dose of the RV vaccine respectively^21^. Studies in Australia, United States, England and nationwide registers in Finland evaluated the association between RV vaccination and IS and found that the benefits of RV immunisation programme outweigh the small increased risks of intussusception^11,22–25^. Furthermore, the post- licensure data in Mexico, Brazil, Australia and the United States showed that 7000 to 69, 600 diarrhoea hospitalisations were prevented by RV vaccination compared to the six to 166 low risk IS cases in infants^10^. However, an analysis to assess the potential benefits of giving the first dose of RV vaccine to infants up to a year of age rather than 12 weeks showed a mortality reduction of 54,087 RV-associated deaths but cause an additional 1,226 IS deaths^26^.

If an IS case temporally associated to RV vaccine case were to occur in Fiji, we found that IS outcomes and clinical care to be excellent. Hydrostatic reduction has been available in Fiji in more recent years. However children often present very late and therefore hydrostatic therapy can be unsuccessful. Surgical intervention is still the most common treatment. Laparoscopy is currently not available in Fiji. Data on partial bowel obstruction was not collected. The access to care is good and there were no IS case fatalities. Deaths associated with IS in developed countries are rare amongst infants and children. In contrast, studies in Africa showed that the mortality rate due to IS was mostly high^27^. A review of Zambian hospitals of IS in children <24 months old showed a high CFR of 33.7%^28^. In Rwanda, delays in presentation and treatment of IS resulted in the 28% mortality rate^29^. Overall, in African countries, the delayed presentation was common and 87% of cases required surgical intervention with a high CFR of 10 to 33.7%^30,31^.

There are limitations to this study. The pre-RV vaccine study was a retrospective review, which is typically prone to bias as the data collected were limited to the register availability and complete recording in the medical records. For the active surveillance, there was delay in reporting of some cases. As such, the vaccination status of all cases was not ascertained. This study was not able to compare the epidemiology before and after the introduction of the RV vaccine due to the small population in Fiji and therefore few IS cases. Any small change in IS cases due to chance would change incidence rates substantially.

In conclusion, we found the baseline epidemiology of IS in Fiji to be similar to other countries, apart from countries in Asia. It is unlikely a temporal association occurred for any IS case in the three year post vaccination period. Nevertheless, we estimated one to five additional IS cases every five years in association with the RV vaccine in Fiji. If a temporally associated vaccine-related IS case was to occur, quality health care is available in Fiji and therefore the benefits of RV vaccination outweigh the risks.

## Methods

### Study site

Fiji is the third largest Pacific Island country. The population was 837,271 in the last census in 2007. The population comprises of 60% indigenous (iTaukei), 35% Fijians of Indian descent and 5% of other ethnicity^32^. The overall annual population growth rate is 0.7% and 1.7% for the indigenous (iTaukei) population and −0.7% for the Fijians of Indian descent^32^. In 2014 and 2015, the under-five mortality rate was 18 and 17 per 1000 live births respectively^33^. There is good access to healthcare facilities and services are provided free of charge. Three tertiary hospitals and 18 sub divisional hospitals have admission facilities to cater for the public. The primary treatment option for IS in Fiji is surgery. Any child needing surgery is referred to their nearest tertiary hospital and admitted for medical care by the paediatricians, with consultation by the surgeons. It is unlikely for a child to die at home from an undiagnosed condition as all home deaths are handled by police and autopsies are undertaken in all cases unless there is a known underlying medical condition. Hospitalisation data are entered into the national hospital admission database by medical coders, using the International Statistical Classification of Diseases and Related Health Problems, Tenth Revision, Australian Modification (ICD-10-AM), and stored at the MoHMS Health Information, Research and Analysis Unit (HIRAU). An audit of the national hospital admission database showed it captured 83% of admissions^34^. National population denominators and growth rates were sourced from Fiji Government Bureau of Statistics, 2007 National Census. The 2007 national population for children under two years of age was 22,176, with 11,265, 9013 and 1898 for the iTaukei, Fijians of Indian descent, and people of other ethnicity, respectively. Population figures were adjusted using ethnicity specific population growth rates^32^. The RV vaccine (Rotarix, GSK) was introduced on 28^th^ October 2012. Infants received RV vaccine as a two dose schedule, given at six and 14 weeks of age. No RV vaccine was available privately prior to national introduction in 2012. The vaccine coverage estimated by the MoHMS for two doses of RV vaccine was 88%, 84% and 89% in 2013, 2014 and 2015, respectively, and the vaccine coverage for one dose of RV vaccine was 91%, 90% and 92% in 2013, 2014 and 2015, respectively. Vaccination data was recorded on the child health card and the health centre registers at the time of vaccination as part of the routine MoHMS data collection.

### Data collection

#### Retrospective review of IS

To describe the epidemiology of IS, potential IS cases aged <24 months old admitted to the paediatric departments of any of the three tertiary hospitals between January 2007 to 28^th^ October 2012, or had IS as their cause of death in the death and autopsy registers were included. Potential IS cases include the following discharge diagnoses: IS, bowel obstruction, intestinal obstruction, and paralytic ileus. Potential IS cases were also sourced from the national electronic hospital patient database (PATIS), according to ICD10-AM codes K56.0-K56.7 as the primary diagnosis. All medical records for potential IS cases were reviewed by a paediatric doctor (ET) and demographic and clinical details were extracted and recorded onto study-specific case investigation forms for each potential IS case. Clinical detail were then reviewed by the paediatric doctor (ET) and classified according to the level 1 Brighton Collaboration definition^35^.

#### Active surveillance of IS following RV vaccine introduction

To determine the temporal relationship of RV vaccine to IS, active IS surveillance was established in the paediatric departments of the three tertiary hospitals from January 2013 to December 2015, following RV vaccine introduction. Paediatricians identified cases of suspected IS and completed the case investigation forms. Potential cases were classified using the Brighton Collaboration criteria^36^ by the paediatric doctor (ET). Dates of vaccination were obtained from the individual child immunisation record and the maternal and child health clinic registers. The date of vaccination is recorded at the time of vaccination on both of these written sources. An expert advisory committee comprised of senior public health official and paediatricians were established to review each IS case and identify if there was any potential RV vaccine association. A vaccine related IS cases was defined as potentially vaccine related: occurring 1–7 days after the first dose of RV vaccine; likely vaccine related: occurring 8–21 days after the first dose of RV vaccine or 1–7 days after the second dose of RV vaccine; or unrelated to the vaccine: occurring 21 days after any dose of RV vaccine^11^.

### Data analysis

The period between January 2007 and October 2012 was defined as the pre-RV vaccine period and the period between January 2013 and December 2015 was defined as the post vaccine period. Data was double entered into an Epidata, version 3.1 (The EpiData Association, JM Lauritsen) database. Non-parametric continuous data were described as the median and interquartile range (IQR), and categorical data were described as frequencies and percentages. The incidence rates were calculated as per 100,000 population in the pre-RV vaccine period for all infants, using STATA, version 14 (Stata Corporation, College Station, TX). In the post-RV vaccine period cases were classified by their temporal association to the vaccine and the median number of days (and IQR) of the IS event post their last dose of RV vaccine. The number of expected additional IS cases following RV vaccine introduction from 2013–2015 was calculated by multiplying the population at risk (infants vaccinated) by the expected rate increase of 1–6 × 10^−5^ (1–6 IS cases per 100,000 population).

Ethical approval was granted from the Fiji National Health Research Ethics Review Committee (approval number; FNRERC 2013 14) before data was collected. All research was performed in accordance with relevant guidelines/regulations. Informed consent was not required by the ethics committee due to the public health importance of the study.

### Data availability

All data generated or analysed during this study are included in this published article.
